# 3D cultivation of non-small-cell lung cancer cell lines using four different methods

**DOI:** 10.1007/s00432-024-06003-x

**Published:** 2024-10-23

**Authors:** Karina Malmros, Nadi Kirova, Heike Kotarsky, Daniel Carlsén, Mohammed S.I. Mansour, Mattias Magnusson, Pavan Prabhala, Hans Brunnström

**Affiliations:** 1 Department of Clinical Sciences Lund, Division of Pathology, Lund University, BMC B13, Klinikgatan 26, Lund, SE-221 00 Sweden; 2 Department of Genetics, Pathology, and Molecular Diagnostics, Skåne University Hospital Lund, Lund, SE-221 85 Sweden; 3 Department of Pathology and Cytology, Halland Hospital Halmstad, Halmstad, SE-301 85 Sweden; 4 Division of Molecular Medicine and Gene Therapy, Lund Stem Cell Center, Lund University, Lund, SE-221 00 Sweden

**Keywords:** Adenocarcinoma, Culture, Immunohistochemistry, Squamous cell carcinoma, Tumoroid

## Abstract

**Purpose:**

The aim of this study was to set up reliable and reproducible culture conditions for 3D tumoroids derived from non-small cell lung cancer (NSCLC) cell lines to enable greater opportunity for successful cultivation of patient-derived samples.

**Methods:**

Four NSCLC cell lines, two adenocarcinomas (A549, NCI-H1975) and two squamous cell carcinomas (HCC-95, HCC-1588), were first cultured in traditional 2D settings. Their expected expression profiles concerning TTF-1, CK7, CK5, and p40 status were confirmed by immunohistochemistry (IHC) before the generation of 3D cultures. Tumoroids were established in the hydrogel GrowDex^®^-T, Nunclon™ Sphera™ flasks, BIOFLOAT™ plates, and Corning^®^ Elplasia^®^ plates. Western blot was used to verify antigen protein expression. Hematoxylin-eosin staining was used to evaluate the cell morphology in the 2D and 3D cultures. Mutational analysis of *KRAS* and *EGFR* by PCR on extracted DNA from 3D tumoroids generated from cells with known mutations (A549; *KRAS* G12S mutation, NCI-H1975; *EGFR* L858R/T790M mutations).

**Results:**

We successfully established 3D cultures from A549, NCI-H1975, HCC-95, and HCC-1588 with all four used cultivation methods. The adenocarcinomas (A549, NCI-H1975) maintained their original IHC features in the tumoroids, while the squamous cell carcinomas (HCC-95, HCC-1588) lost their unique markers in the cultures. PCR analysis confirmed persistent genetic changes where expected.

**Conclusion:**

The establishment of tumoroids from lung cancer cell lines is feasible with various methodologies, which is promising for future tumoroid growth from clinical lung cancer samples. However, analysis of relevant markers is a prerequisite and may need to be validated for each model and cell type.

**Supplementary Information:**

The online version contains supplementary material available at 10.1007/s00432-024-06003-x.

## Introduction

Lung cancer is the most common cause of cancer-related deaths worldwide (Global Burden of Disease Cancer et al. 2019). In the last decades, novel treatment strategies such as targeted therapy and immunotherapy have been introduced. Although the prognosis has improved for lung cancer, the survival rate is still limited, and further therapies are needed (Siegel et al. 2024). Development of new anti-cancer drugs takes time, and many substances that show promising results in vitro do not result in approved drugs for clinical use, often due to limited effect in vivo (Harrison 2016; Dowden and Munro 2019). Improvement of pre-clinical in vitro models for drug screening is one possibility to address this problem, and tumoroid culturing is promising for this purpose (Rios and Clevers 2018).

Tumoroids (also called cancer organoids) are patient-derived tumor cells or cell lines growing in three-dimensional (3D) structures supported by the addition of e.g. a substrate enabling such growth. The method has been used in the field of lung cancer (Werner et al. 2021; Lee et al. 2021; Koga et al. 2023; Rossi et al. 2022), but overgrowth by benign cells from airways and lung parenchyma is a challenge for patient-derived lung tumoroids (Dijkstra et al. 2020). Morphology, immunohistochemistry (IHC), and analysis of genetic alterations may be useful in the determination of the presence and proportion of malignant cells in tumoroids (Dijkstra et al. 2020; Werner et al. 2021; Koga et al. 2023).

Lung cancer is broadly divided into non-small cell lung cancer (NSCLC) and small cell lung cancer (The WHO 2021). The former is a heterogeneous group comprising 80–85% of lung cancer cases, with adenocarcinoma and squamous cell carcinoma being the two most common subtypes. Lung adenocarcinomas express cytokeratin 7 (CK7) and in most cases thyroid transcription factor-1 (TTF-1), where the latter is considered lineage-specific as it is essentially not expressed in squamous cell carcinoma (Vidarsdottir et al. 2018; Kadota et al. 2015; Kriegsmann et al. 2019; Roberts et al. 2020). Correspondingly, squamous cell carcinomas typically express p40 and CK5, whereas these lineage markers are rarely expressed in lung adenocarcinoma (Vidarsdottir et al. 2019; Roberts et al. 2020; Kriegsmann et al. 2019). Furthermore, all the mentioned markers are not expressed in macrophages, lymphocytes, or fibroblasts. However, benign pneumocytes express CK7 and TTF-1, columnar cells from the airways express CK7 and sometimes CK5, and basal cells express p40, CK5, and occasionally TTF-1. If morphology is not obvious, the detection of tumor-specific genetic alterations may be of aid in establishing the presence or fraction of tumor cells. Thus, it is important that in vitro models do not significantly affect diagnostic IHC and molecular methods used for separating benign epithelial cells from tumor cells.

The 3D substrates used for lung cancer tumoroid culturing in recent publications have essentially been growth factor-reduced basement membrane matrices like Matrigel^®^, Cultrex™, or Geltrex™ (Werner et al. 2021; Koga et al. 2023; Rossi et al. 2022; Benton et al. 2009, 2014). The basement membrane extracts derive from Engelbreth-Holm-Swarm (EHS) tumors in mice, which may result in batch-to-batch variability.

The present study aimed to investigate alternative non-animal-based clinically applicable methods to generate tumoroids including GrowDex^®^-T hydrogel, Nunclon™ Sphera™ flasks, BIOFLOAT™ 96 well ultra-low attachment TC plates, and Elplasia^®^ 6 well plates. In this first step, lung cancer cell lines were used to evaluate 3D formation and the substrates’ possible impact on IHC and molecular analysis of the cells that make up the tumoroids.

## Materials and methods

### Cell lines

Nine lung cancer cell lines, whereof three adenocarcinomas (NCI-H1975, A110L, and A549) and six squamous cell carcinomas (SK-MES-1, B1203L, NCI-H520, NCI-H1703, HCC-1588, and HCC-95) were initially included in the project. All cell lines were cultured in Dulbecco’s Modified Eagle’s Medium (DMEM), high glucose (Thermo Fisher Scientific, Waltham, MA, USA) with added 10% fetal bovine serum (FBS) (Thermo Fisher Scientific) in 37 ℃, 5% CO_2_ with > 90% relative humidity in the incubator.

### Immunohistochemistry

Each cell line was characterized regarding its expression (i.e. positivity or negativity) for TTF-1 and CK7, p40 and CK5, or all four markers with IHC on 3–4 micrometer thick sections from paraffin-embedded cell blocks after formalin fixation. The antibodies used were TTF-1 clone 8G7G3/1, (Roche Diagnostics, Indianapolis, IN, USA, #05479312001, ready-to-use), CK7 clone SP52 (Roche Diagnostics, #05986818001, ready-to-use), p40 clone BC28 (Roche Diagnostics, #05867061001, 1:50), and CK5 clone XM26 (Leica Biosystems, Newcastle, UK, NCL-L-CK5, 1:25). All staining was automatically performed on a Ventana BenchMark Ultra after pre-treatment with cell conditioning 1 (EDTA, pH 8) at the Department of Genetics, Pathology, and Molecular Diagnostics, Skåne University Hospital Lund, in accordance with our previous studies on lung cancer material (Vidarsdottir et al. 2018, 2019) and clinical routine samples. For TTF-1 and p40, nuclear positivity was evaluated, while for CK7 and CK5 cytoplasmic positivity was assessed. Control tissue was used on each slide; thyroid (positive), tonsil, and placenta for TTF-1, liver (bile ducts positive), tonsil (epithelium positive), and appendix for CK7, tonsil (epithelium positive), prostate (basal cells positive), appendix, and placenta for p40 and CK5.

### 3D cultures

Based on the expression of relevant antigens by IHC in the 2D cultures, selected cell lines were cultured in 0.2% GrowDex^®^-T hydrogel (UPM Biomedicals, Helsinki, Finland), Nunclon™ Sphera™ flasks (Thermo Fisher Scientific), BIOFLOAT™ 96 well ultra-low attachment TC plates (Sarstedt, Nümbrecht, Germany), or Elplasia^®^ 6 well plates (Corning via Saveen & Werner, Malmö, Sweden) and monitored for tumoroid generation. The growing tumoroids were monitored at least thrice a week and fresh medium (DMEM, high glucose, with 10% FBS) was added when needed. After two weeks the formed tumoroids were analyzed for antigen expression using IHC (as described above), complemented with Western blotting to assess the conservation of expected proteins and mutation analysis (see below). The tumoroids grown in the hydrogel GrowDex^®^-T were released by addition of the GrowDase™ enzyme (UPM Biomedicals, #900 102 002), which degrades the starch fibers in a process taking approximately 8 h, prior to the analyses.

### Western blot

A sample solution was prepared using β-mercaptoethanol added to 2x Laemmli Sample Buffer (Bio-Rad Laboratories, Hercules, CA, USA) in a 1:20 proportion. The pelleted tumoroid cells were resuspended in the sample buffer solution and incubated in a heat block at 95 ℃ for 5 min. Samples were cooled to 4 ℃ and centrifuged at 15 000 x g for 2 min, whereafter 10 µl of supernatant from each sample was transferred to a Mini-PROTEAN^®^ TGX™ Precast Gel (Bio-Rad Laboratories) along with 5 µl of Proteintech Prestained Protein Marker 10–180 kDa (Proteintech, Rosemont, IL, USA; #PI00001) or Invitrogen™ Novex™ Sharp Pre-stained Protein Standard 3.5–250 kDa solution (Thermo Fisher Scientific) as a gradient marker. Gel electrophoresis was conducted at 150 V for 45 min submerged in tris-glycine-sodium dodecyl sulfate (TGS) running buffer. Bands were transferred to a Trans-Blot^®^ Turbo™ Mini 0.2 μm PVDF Transfer Pack (Bio-Rad Laboratories) and processed at 25 V for 7 min. PVDF sheets were submerged in EveryBlot Blocking Buffer (Bio-Rad Laboratories, #12010020) for 30 min, then washed in the same buffer for 5 min and incubated in 10 ml primary antibody-blocking buffer solution overnight at 4 ℃. The antibodies used were TTF-1 monoclonal antibody (Proteintech, #66034-1-Ig) diluted 1:1400 and monoclonal Cytokeratin 7, CK7 (Proteintech, #66483-1-Ig) diluted 1:20 000. The expected molecular weight is 42 kDa for TTF-1 and 51 kDa for CK7. Horse-Radish-Peroxidase conjugated Beta actin mouse McAb (Proteintech, #HRP-66009) was used as a loading control for the protein content in the wells.

After incubation, the PVDF sheets were washed in 7 ml blocking buffer for 5 min, repeated twice, and incubation with the secondary antibody Goat Anti-Mouse IgG (H + L)-HRP (Bio-Rad Laboratories, #170 − 65) diluted 1:5000 was performed in room temperature for 1.5 h (except for the HRP-conjugated mouse McAb), after which a washing step in EveryBlot Blocking Buffer, 2 × 5 min was conducted. The PVDF sheets were then soaked in a 1:1 mix of TS Luminoid/Enhancer solution and Stable Peroxide solution (Bio-Rad Laboratories) for 1 min. The washing step in the blocking buffer was repeated and then the membranes were analyzed in a ChemiDoc^™^ MP Imaging System (Bio-Rad) using its chemiluminescence and calorimetric settings. The Image Lab analysis software 6.1 was used to capture and analyze the detected protein bands on the membranes.

### Mutation analysis

DNA was extracted from adherent 2D cultures and 3D tumoroids using QIAamp^®^ DNA Blood Mini Kit (QIAGEN^®^, Hilden, Germany) according to the manufacturer’s protocol. To extract enough DNA from the tumoroids grown in GrowDex^®^-T, an enzymatic reaction with GrowDase™ (UPM Biomedicals, #900 102 002) at a working concentration of 1800 µg/mg, following instruction from the manufacturer, had to be performed with incubation overnight at 37 ℃. Also, an additional step with recovery of the released tumoroids had to be introduced, where the cell cultures were diluted in 30 ml of Dulbecco′s Phosphate Buffered Saline (DPBS; Thermo Fisher Scientific), followed by centrifugation at 2500 x g for 5 min, whereafter the supernatant was removed and 1.2 ml RLT buffer (QIAGEN, #79216) added to lyse the cells in the GrowDex^®^-T cultures. For the other tumoroid growing methods and 2D cultures, the cells were pelleted at 900 x g for 5 min before DNA extraction. Eluted DNA was quantified with the QuantiFluor^®^ dsDNA System in a Quantus™ Fluorometer (Promega Biotech, Madison, WI, USA).

To evaluate the persistence of known *EGFR* and *KRAS* mutations in NCI-H1975 and A549 cells, respectively, the Idylla™ KRAS Mutation Test (Biocartis NV, Mechelen, Belgium, #A0020/6) and the Therascreen EGFR RGQ PCR Kit (QIAGEN, #874111) were used in accordance with the instructions for the kits and clinical routine at the Department of Genetics, Pathology, and Molecular Diagnostics, Skåne University Hospital Lund. Further details may be provided upon request. The DNA input in the analyses was 80–250 ng and all samples fulfilled the criteria for the quality control.

## Results

### Cell line selection

**Fig. 1:**
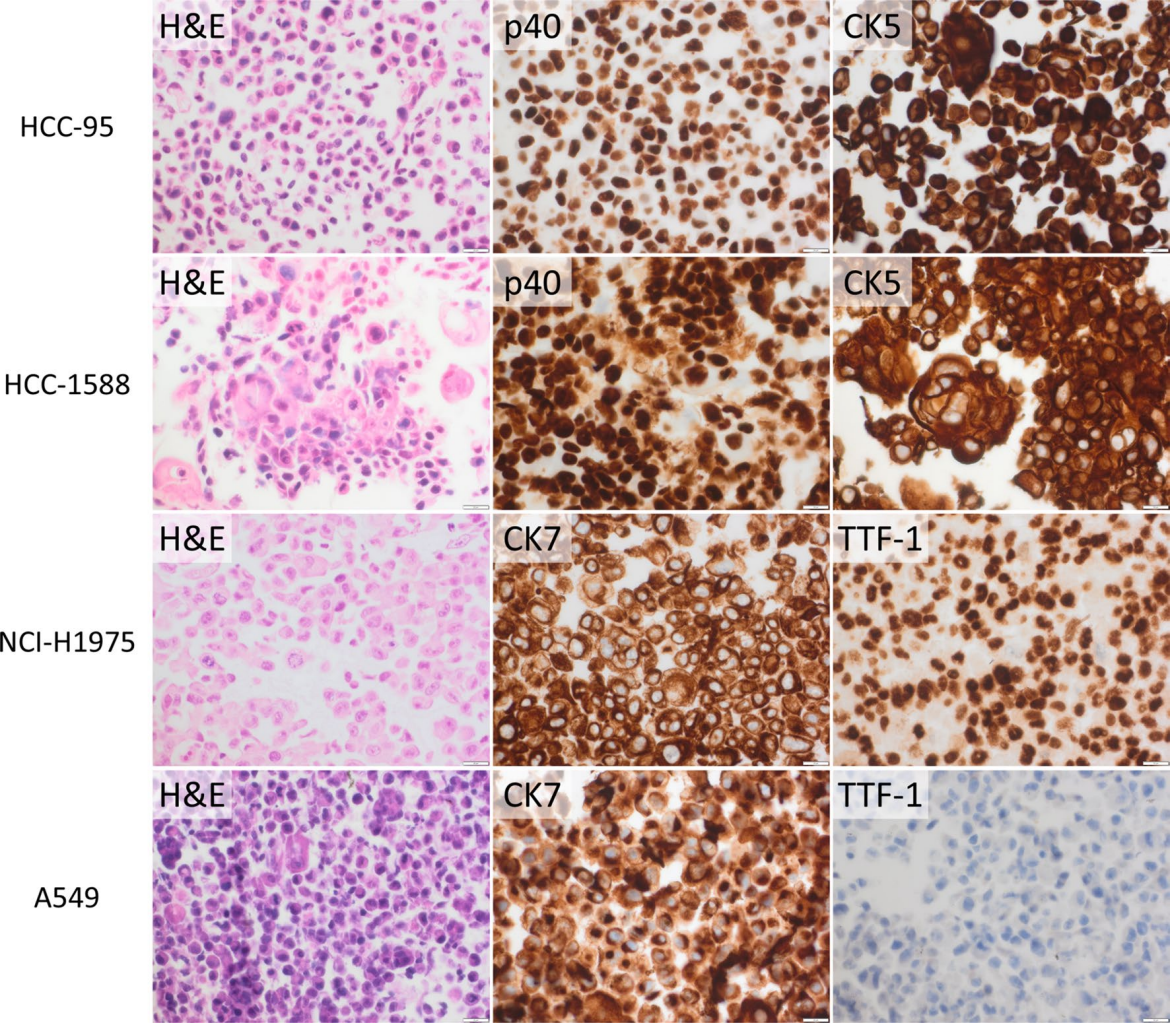
Protein expression evaluated with immunohistochemical (IHC) analysis for squamous (p40, CK5) and adenocarcinoma (CK7, TTF-1) markers together with hematoxylin-eosin (H&E) staining of the selected cell lines in 2D culturing. Note (atypical) nuclear CK7 staining in some A549 cells. The scale bar is 20 μm

The lung cancer cell lines in the 2D cultures were initially characterized with IHC on sections from cell blocks. CK5 and p40 were positive in the squamous cell cancer cell lines HCC-95 and HCC-1588 (see Fig. 1), while SK-MES-1, B1203L, NCI-H520, and NCI-H1703 were negative for CK5 and p40 (occasional NCI-H520 cells, < 1%, expressed CK5 only, see Supplementary Fig. 1, also including images of control tissue). Regarding the adenocarcinoma cell lines, NCI-H1975 was positive for both TTF-1 and CK7, while A549 and A110L were positive only for CK7. Positive cases exhibited diffuse and strong positivity (Fig. 1) except A110L which only showed moderate CK7 positivity (Supplementary Fig. 1). Consequently, only three of the nine evaluated cell lines displayed lung cancer lineage-specific expression. Apart from HCC-95, HCC-1588, and NCI-H1975 the cell line A549 was also selected for 3D culturing based on the known *KRAS* mutation.

The squamous cell lines included in the further analyses (HCC-95 and HCC-1588) were also stained for the markers commonly expressed in adenocarcinomas (CK7 and TTF-1) and the selected adenocarcinoma cell lines (NCI-H1975 and A549) were stained for squamous markers (p40 and CK5). HCC-95 exhibited a moderate CK7 expression, while for HCC-1588 and NCI-H1975 few cells expressed CK7 and p40, respectively, see Supplementary Fig. 2.

### 3D tumoroid cultures

Expanded trials with 0.2% GrowDex^®^-T-containing growth medium solution, Sphera™ flasks, BIOFLOAT™ 96 well plates, and Elplasia^®^ 6 well plates displayed successful tumoroid generation within 24 h with each method for all selected lung cancer cell lines (HCC-95, HCC-1588, NCI-H1975, and A549). In the initial arrangement into tumoroid formation the cells were loosely organized, though HCC-95 and HCC-1588 developed a clear border within 72 h while NCI-H1975 and A549 instead formed a denser core surrounded by a sparser outer layer.

**Fig. 2:**
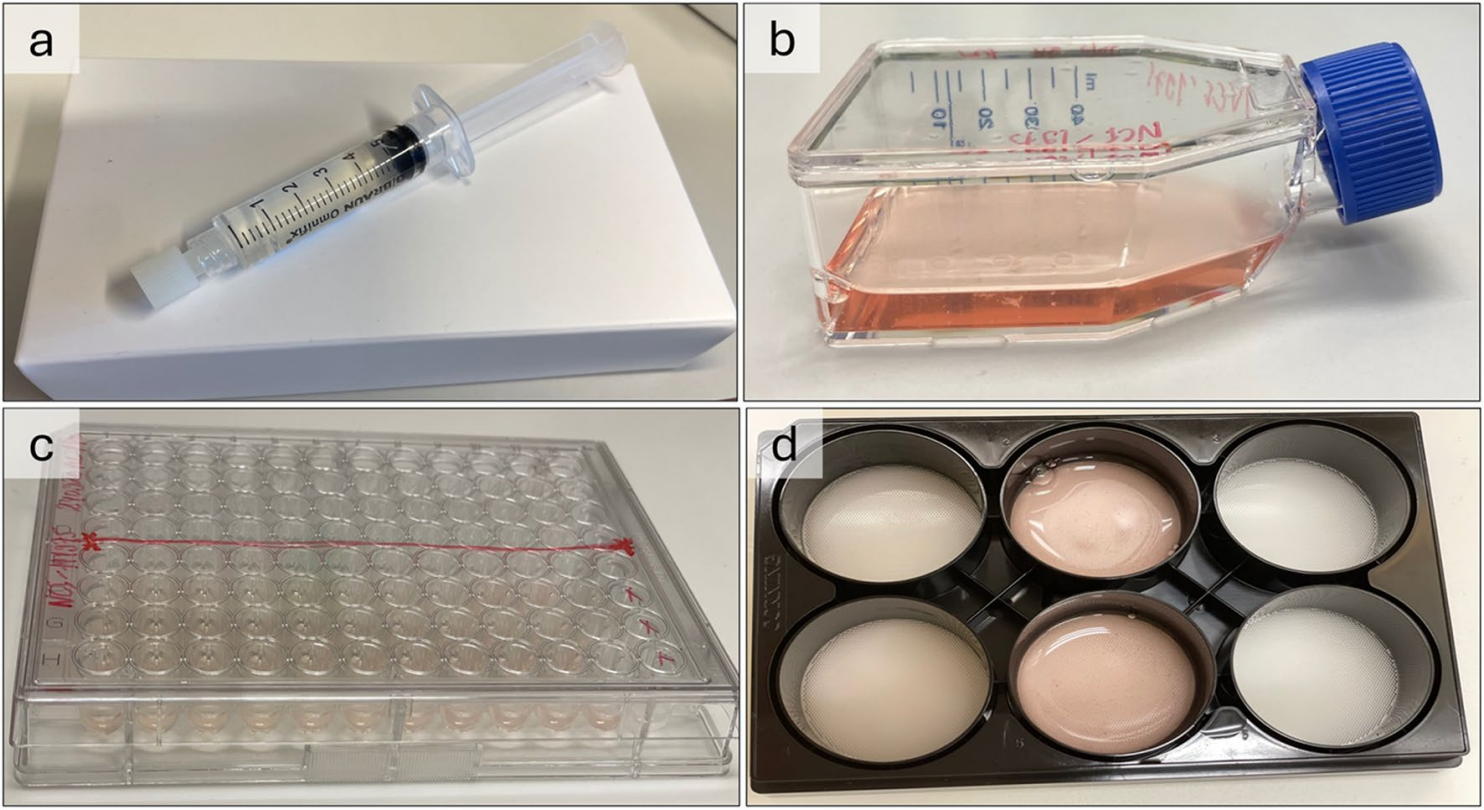
The four different 3D culturing methods used. **(a)** GrowDex^®^-T, a transparent hydrogel in its original 1.0% stock solution delivered in a syringe **(b)** Nunclon™ Sphera™ flask, **(c)** BIOFLOAT™ 96 well plate, and **(d)** Elplasia^®^ 6 well plates

**Fig. 3:**
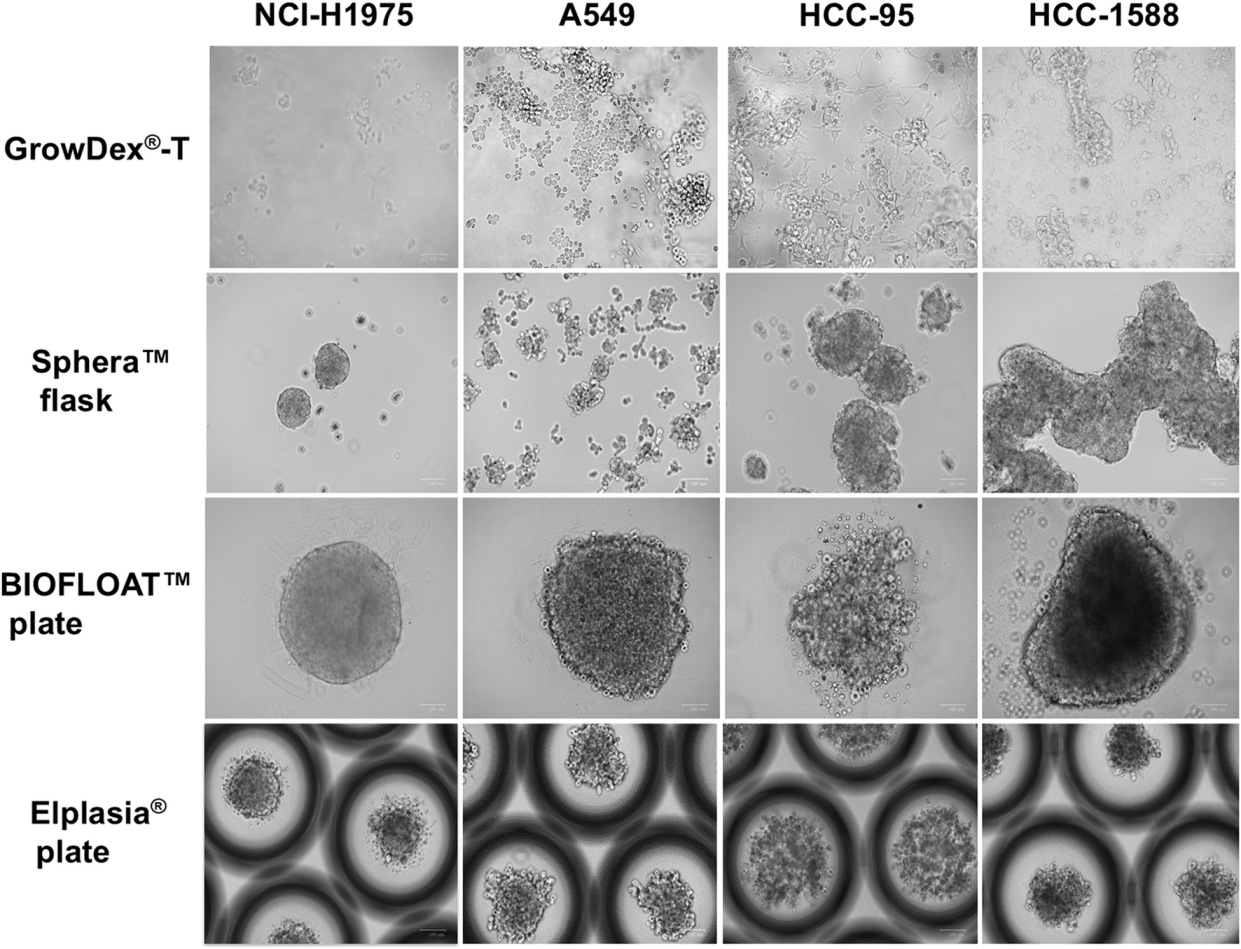
Images of the tumoroids generated from the cancer cell lines NCI-H1975, A549, HCC-95, and HCC-1588

The tumoroids in the GrowDex^®^-T solution established themselves on multiple levels in the matrix, with an increase to large-size clusters observed on day 5. In the BIOFLOAT™ 96 well plates, the cells showed rapid formation of cell clusters within 8 h, and in the 6 well Elplasia^®^ plates growing tumoroids were identified in the microcavities, often distributed with a sole tumoroid per microcavity. Lastly, for the Sphera™ flasks, a similar growth pattern was noted in all cases, with an initial formation of small tumoroids that later converged into larger clusters. Images of the methods used to produce 3D tumoroids from the lung cancer cell lines are shown in Fig. 2, while images of the growing tumoroids are shown in Fig. 3.

Overall, HCC-95 showed the fastest growth while NCI-H1975 displayed the slowest (no exact quantification was attempted). The tumoroid cultures were maintained for approximately two and a half weeks before the trials were terminated.

### Protein expression analysis

**Fig. 4:**
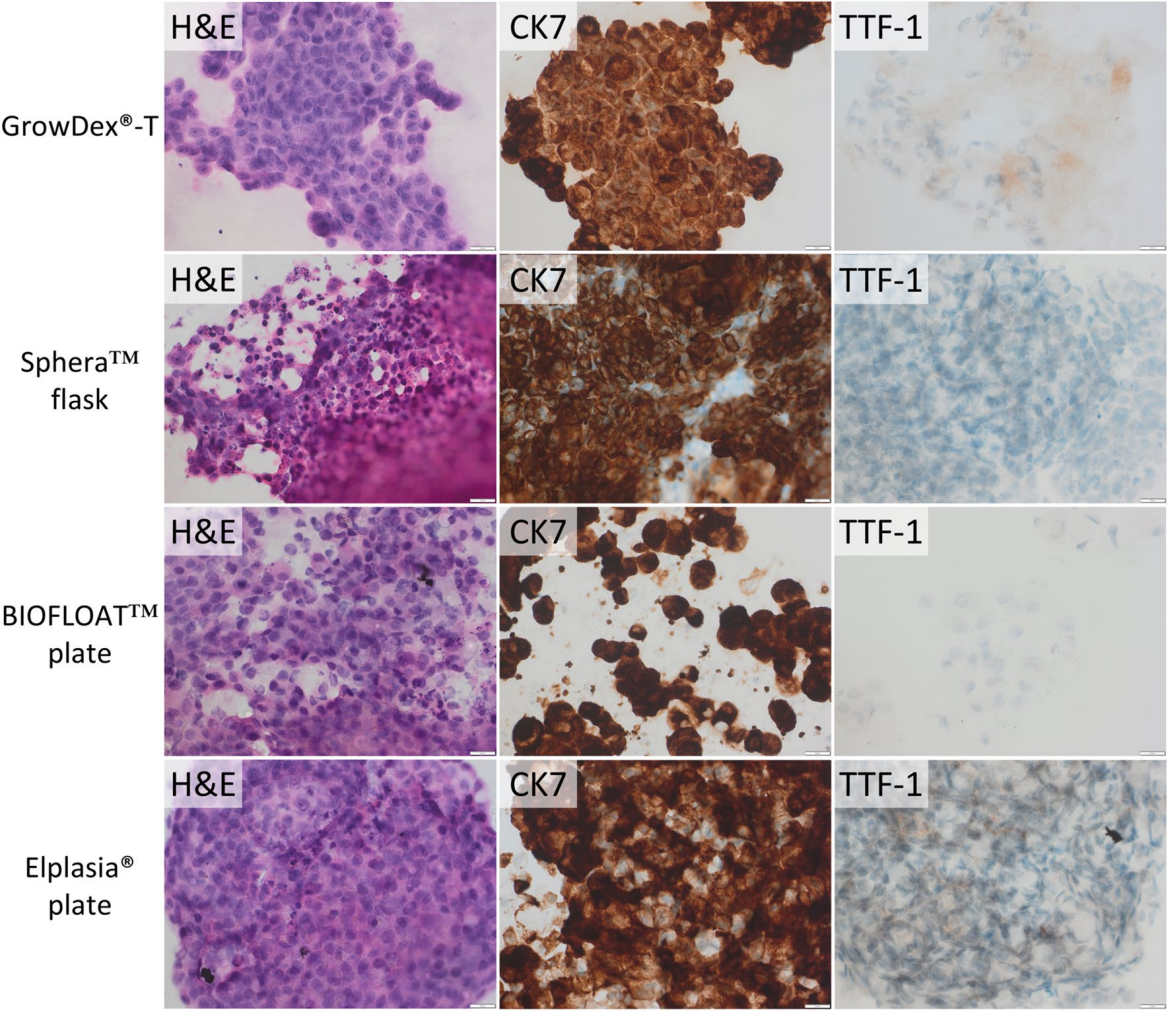
Tumoroids generated from A549 cells in GrowDex^®^-T hydrogel, Nunclon™ Sphera™ flask, BIOFLOAT™ 96 well plate, and Corning^®^ Elplasia^®^ 6 well plate. Staining with hematoxylin-eosin (H&E) and IHC using CK7 and TTF-1 antibodies. The scale bar is 20 μm

**Fig. 5:**
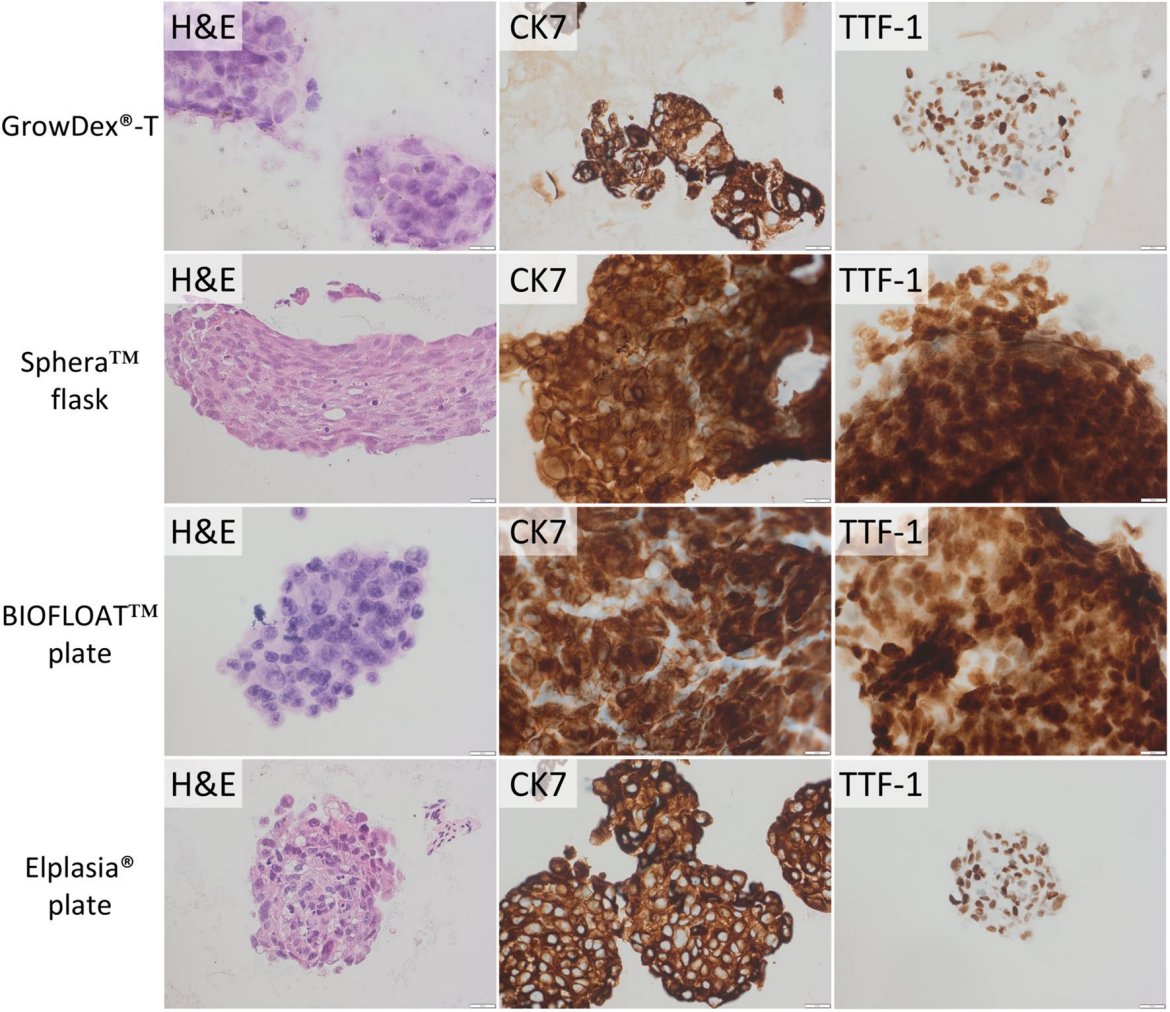
Tumoroids generated from NCI-H1975 cells in GrowDex^®^-T hydrogel, Nunclon™ Sphera™ flask, BIOFLOAT™ 96 well plate, and Corning^®^ Elplasia^®^ 6 well plate. Staining with hematoxylin-eosin (H&E) and IHC using CK7 and TTF-1 antibodies. The scale bar is 20 μm

After 3D culturing, protein expression for TTF-1 and CK7 was evaluated using IHC on cell block sections for the cell lines A549 and NCI-H1975, while p40 and CK5 were assessed for HCC-95 and HCC-1588. Images from A549 and NCI-H1975 cells cultured in 3D are presented in Figs. 4 and 5, respectively. As evident from the figures, the adenocarcinoma cell lines retained their expression profiles with positive CK7 and positive CK7 and TTF-1, respectively, in all culturing methods. In contrast, p40 and CK5 were negative for both HCC-95 and HCC-1588 for the 3D culturing methods selected for analysis (images in Supplementary Fig. 3). The control tissue stained normally for these analyses, and the IHC staining was repeated with the same results. Follow-up staining of HCC-95 and HCC-1588 cultured in only 2D for several weeks also showed loss of protein expression using IHC for p40 and CK5 (Supplementary Fig. 4).

**Fig. 6:**
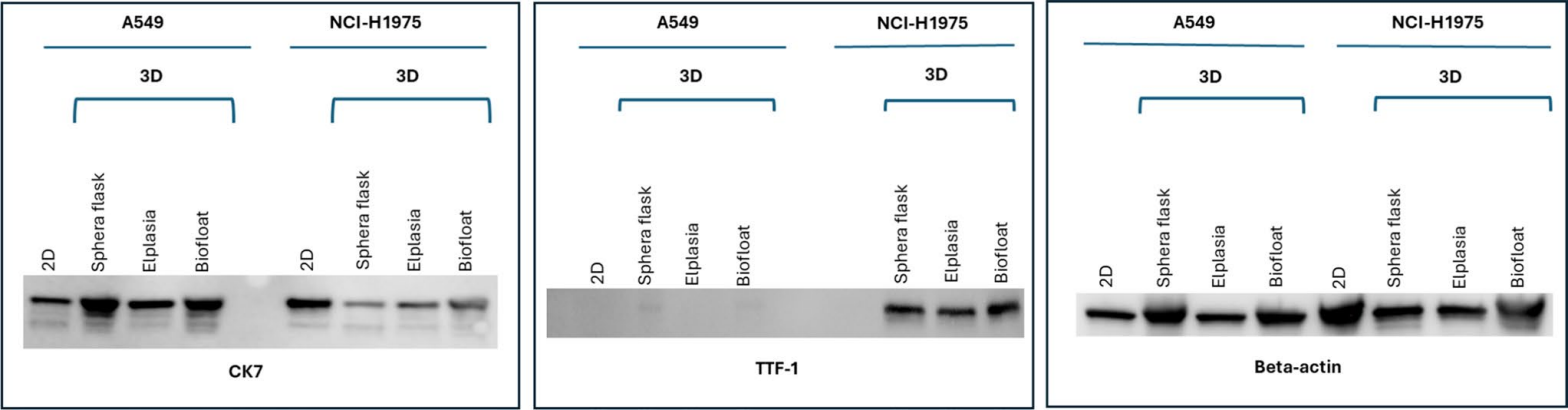
Western blot identifying CK7 (both A549 and NCI-H1975) and TTF-1 (NCI-H1975) protein expression in 2D and selected 3D cultures. Beta-actin was used as endogenous control showing the protein content in the same sample preparations used to be loaded on the gels

Western blot results for the adenocarcinoma cell lines A549 and NCI-H1975 confirmed the protein expression identified by IHC, as shown in Fig. 6.

The IHC expression of markers commonly positive in adenocarcinomas (CK7 and TTF-1) in the squamous cell lines (HCC-95 and HCC-1588) and squamous markers (p40 and CK5) in the adenocarcinoma cell lines (NCI-H1975 and A549) were investigated for selected 3D substrates and is presented in Supplementary Figs. 5 and 6, respectively. A moderate CK7 expression was seen for both squamous cell lines (i.e. somewhat higher than in 2D cultures for HCC-1588) and occasional NCI-H1975 cells were consistently positive for p40 in the investigated 3D cultures regardless of substrate.

### Mutation analysis

The original mutational status for A549 (*KRAS* G12S mutation) and NCI-H1975 (*EGFR* L858R/T790M mutations) was confirmed in these two cell lines for all 3D culturing methods GrowDex^®^-T hydrogel, Nunclon™ Sphera™ flasks, BIOFLOAT™ 96 well TC plates, and Elplasia^®^ 6 well plates. Selected PCR curves obtained with the Idylla™ KRAS Mutation Test for *KRAS* G12S (c.34G > A; exon 12) and cycle threshold values from the Therascreen EGFR RGQ PCR Kit for *EGFR* L858R (c.2573T > G; exon 21) and T790M (c.2369 C > T; exon 20) are presented in Supplementary Fig. 7 and Supplementary Table 1, respectively.

## Discussion

In the present study, we used four different methods for 3D culturing of lung cancer cell lines, including both adenocarcinoma and squamous cell carcinoma to reflect common NSCLC subtypes. The rapid formation of tumoroids for all cell lines, and the preserved IHC expression and mutation status for the adenocarcinoma cell lines, demonstrate that these 3D methods are feasible approaches. The loss of lineage marker expression for both the squamous cell lines is troublesome. Although it was likely due to biological factors (e.g. dedifferentiation) known to occur in cell lines used for many passages rather than an effect of the 3D methods (supported by the loss of p40/CK5 in extended 2D cultures and the retained CK7 expression in the 3D cultures), it limits our evaluations. Analysis of relevant markers is a prerequisite in patient-derived tumoroids to determine tumor cell content, as overgrowth of benign cells occurs (Dijkstra et al. 2020), and to ascertain preserved similarity with the parental tumor. However, further investigation of the matter was not within the scope of this study, but the findings stress that methods such as IHC and molecular analysis may need to be validated for each model and cell type.

The growth of tumoroids is more similar to tumors in vivo than the use of traditional 2D cultures and is expected to be advantageous as drug screening models (Rios and Clevers 2018; Xu et al. 2018). However, lung cancer tumoroids pose a great challenge, and limited success in reproducibility and feasibility for patient-derived samples has been described (Yokota et al. 2021; Dijkstra et al. 2020; Hynds et al. 2018), although a higher success rate was reported in a recent study (Koga et al. 2023). So far, the growth factor-reduced basement membrane matrices Matrigel^®^, Cultrex™, or Geltrex™ have been used for tumoroid growth (Werner et al. 2021; Koga et al. 2023; Rossi et al. 2022). A disadvantage of these matrices is that they are derived from mice, which causes uncertainty in the biological activities of their components and batch-to-batch variation. Also, these matrices need to be handled at low temperatures to avoid polymerization during handling in the experimental setting. In comparison, the animal-free 3D culture scaffold matrix GrowDex^®^-T can be handled at room temperature and consists of pure nanofibrillar cellulose and water, with no autofluorescence, simplifying imaging and detection. The present study aimed to investigate alternative substrates and methods for 3D culturing, starting with lung cancer cell lines to, in the next step, proceed with patient-derived samples. In our experience, cultivation did not prove demanding concerning the number of practical steps or required equipment for the four methods used, which is why the prospects of clinical application may be promising.

Generation of cell line tumoroids in culture flasks and wells, mimicking the development of solid tumors with homogenous growth, requires minimal cell attachment to prevent adsorption to the surface of the flask or well. The used Nunclon™ Sphera™ flasks, BIOFLOAT™ 96 well TC plates, and Elplasia^®^ 6 well plates have ultra-low attachment surfaces, with a specific cell-repelling surface treatment preventing monolayer cell adhesion to the culture vessel. The preferred method(s) may depend on cell availability (amount of cells), desire, the need for sizable tumoroids, and avoidance of the hypoxic core. We did not investigate all these parameters in the present study, as our focus was protein expression of lineage-markers and driver mutations in the tumoroids. Here, we note that GrowDex^®^-T requires an additional step involving enzymatic release of the tumoroids growing in the matrix, which takes a little more time in the harvesting step compared to the other methods. For a more extensive comparison of potential differences in tumoroid formation and growth between substrates, repeated culturing with ideally patient-derived samples would be needed, which was outside the scope of the present investigation.

A further aspect is the aim of reducing animal-based materials in research. Tumoroids will hopefully prove an important method that may replace at least some animal testing in the development of new anti-cancer drugs and investigations of tumor biology. Also, the methods evaluated in our study are, in contrast to Matrigel^®^, Cultrex™, and Geltrex™, not animal-based products.

Research studies on lung cancer using GrowDex^®^-T, Nunclon™ Sphera™ flasks, BIOFLOAT™ plates, or Corning^®^ Elplasia^®^ plates are difficult to find. GrowDex^®^ (not GrowDex^®^-T as in our investigation) was recently used in two studies on bladder (Walz et al. 2023) and ovarian (Feodoroff et al. 2023) cancer. In the former, the authors concluded that the choice of scaffold may be important given the impact on tumoroid size and gene expression (Walz et al. 2023), while in the latter tumoroid growth of patient-derived cells used for drug sensitivity evaluations was similar for GrowDex^®^ and Matrigel^®^ (Feodoroff et al. 2023). Further, CorningⓇ ElplasiaⓇ plates have been successfully used for growth of hepatocyte spheroids (Kukla et al. 2024). The generation of better experimental cultures for lung organoids has been an issue at stake for more than a decade (Nichols et al. 2014). Here, our investigation may contribute by demonstrating the successful growth of lung cancer cell lines using these methods, but further research is needed.

In conclusion, we were able to establish 3D cultures quickly, reliably, and reproducibly from selected lung cancer cell lines, using four different approaches. These should be further evaluated for tumoroid growth from patient-derived samples.

## Electronic supplementary material

Supplementary Material 1

## Data Availability

No datasets were generated or analysed during the current study.
