# Supplementary material for: 3D cultivation of non-small-cell lung cancer cell lines using four different methods
Source: J Cancer Res Clin Oncol. 2024 Oct 23;150(10):472. doi: 10.1007/s00432-024-06003-x (PMC11499447; doi:10.1007/s00432-024-06003-x)
Supplement: Supplementary file 1 — Supplementary Material 1 [file 432_2024_6003_MOESM1_ESM.pdf]

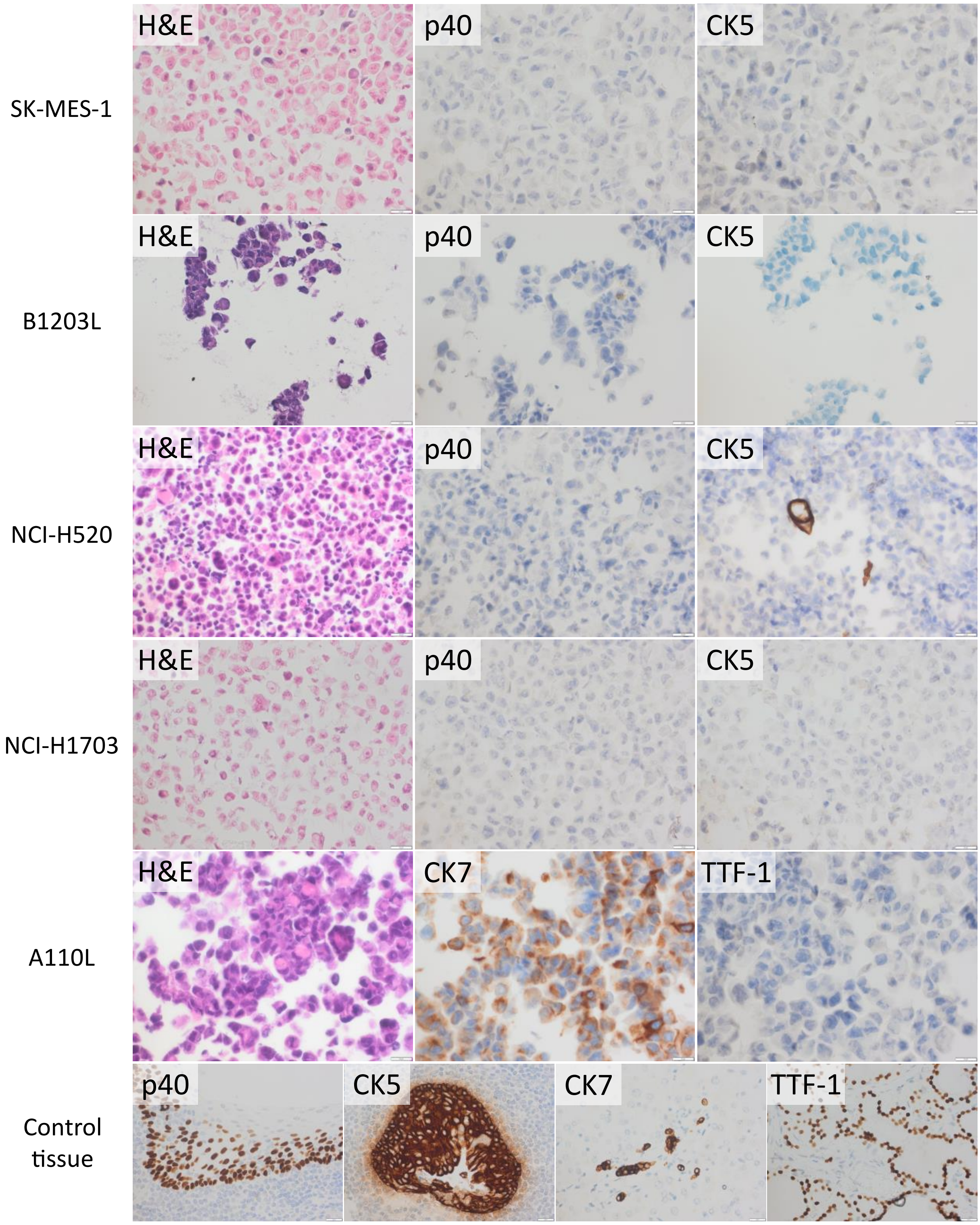

Supplementary Figure 1. Staining of cell block sections after 2D culturing of lung cancer cell lines that were excluded from further analysis due to lack of lineage-marker expression (TTF-1 for adenocarcinoma, and p40 and cytokeratin 5 for squamous cell carcinoma). Staining of control tissue from the slides is included for comparison (benign tonsil for p40 and CK5, liver [bile ducts] for CK7, and thyroid for TTF-1). The scale bar is 20 µm.

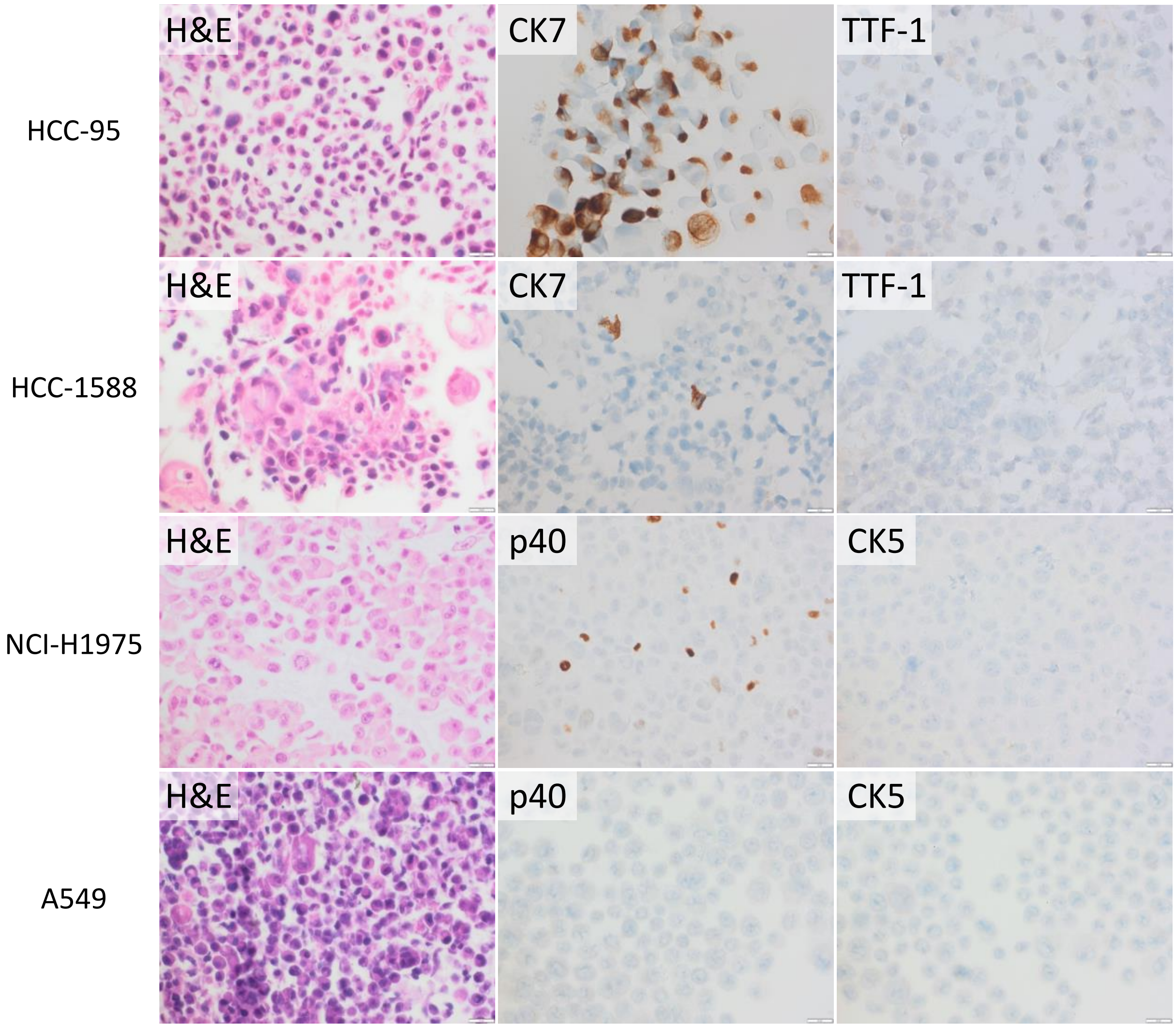

Supplementary Figure 2. Staining of cell block sections after 2D culturing of the included lung cancer cell lines regarding markers commonly expressed in lung adenocarcinomas (CK7 and TTF-1) in the squamous cell lines (HCC-95 and HCC-1588) and squamous markers (p40 and CK5) in the adenocarcinoma cell lines (NCI-H1975 and A549). The scale bar is 20 µm.

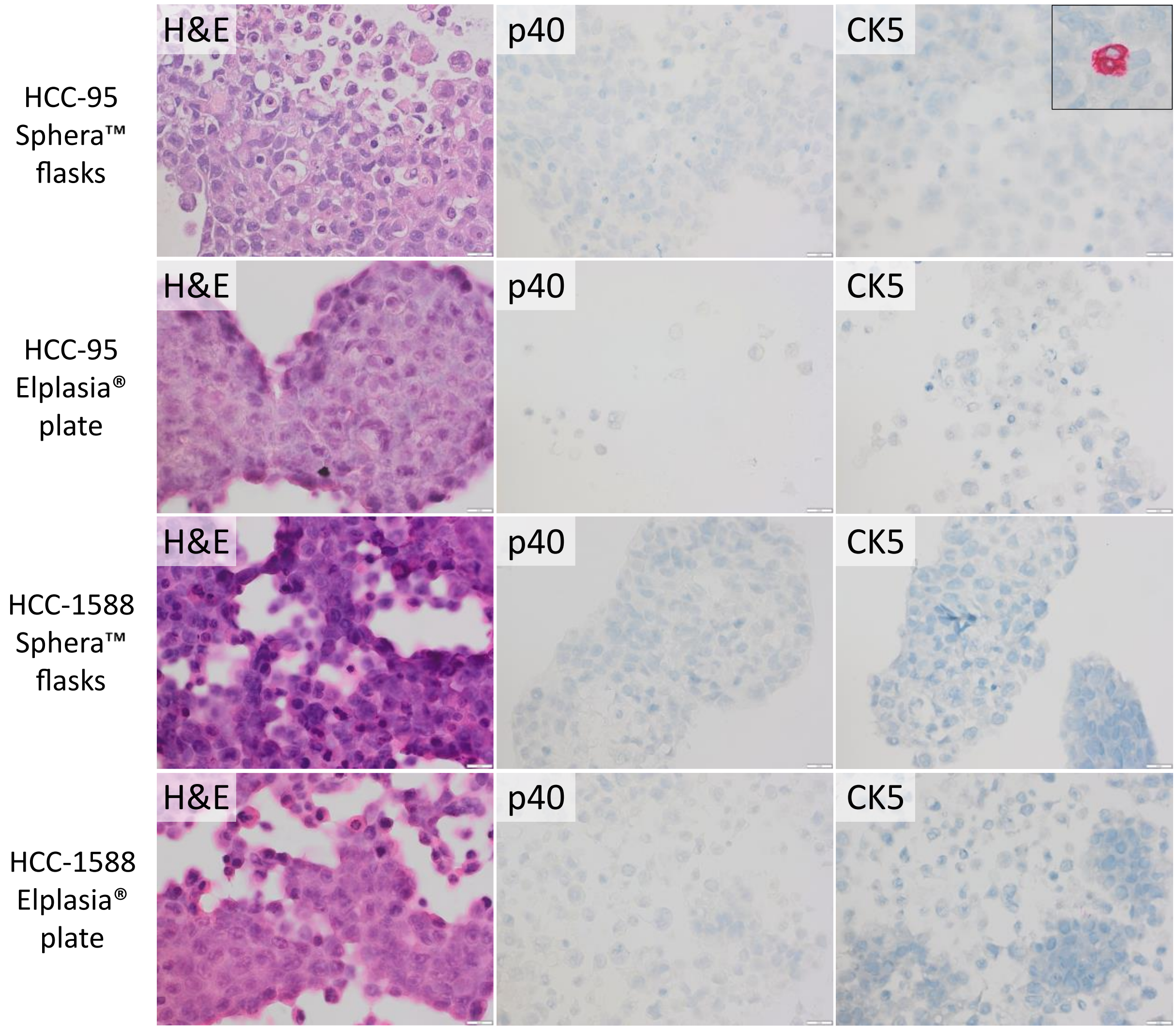

Supplementary Figure 3. Staining of cell block sections after 3D tumoroid culturing in Nunclon™ Sphera™ flasks and Corning® Elplasia® 6 well plates, respectively, of the squamous lung cancer cell lines HCC-95 and HCC-1588 showing negative lineage markers p40 and CK5. Note the occasional positive HCC-95 cell of Sphera™ flask tumoroids in repeated CK5 staining with Red Chromogen (inset). HCC-95 cells from Elplasia® 6 well plates were limited in number in the p40 staining. The scale bar is 20 µm.

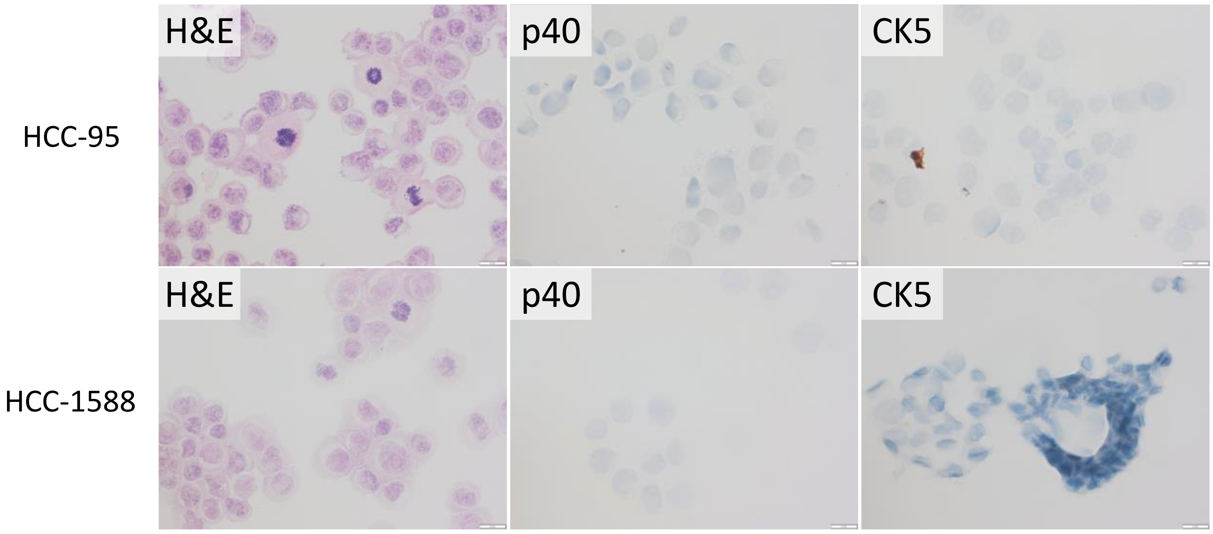

Supplementary Figure 4. Staining of cell block sections after 2D culturing of the squamous lung cancer cell lines HCC-95 and HCC-1588 for several weeks showing loss of lineage markers p40 and CK5. HCC-1588 cells were limited in number in the p40 staining. The scale bar is 20 µm.

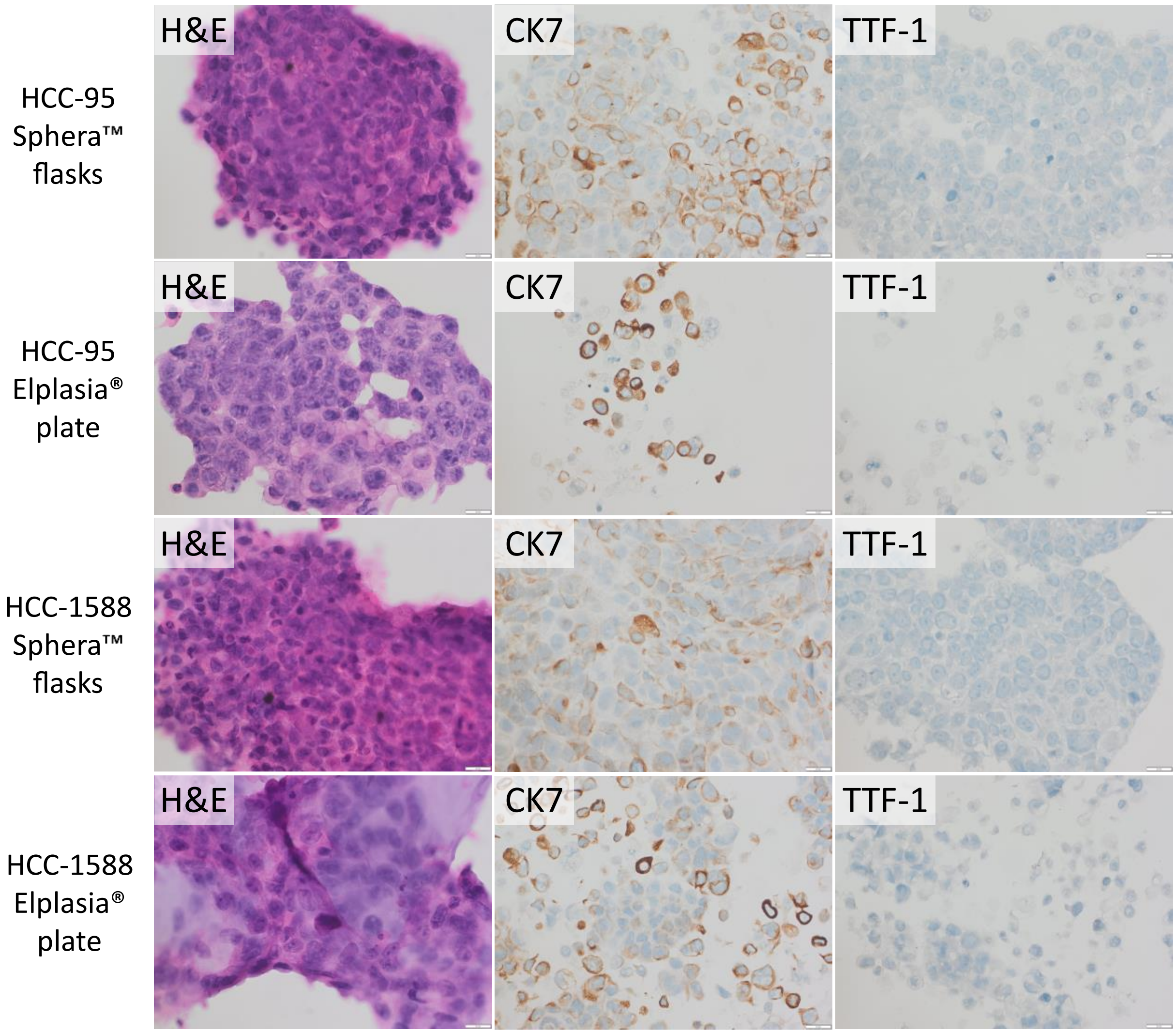

Supplementary Figure 5. Staining of 3D tumoroid cultures from Nunclon™ Sphera™ flasks and Corning® Elplasia® 6 well plates, respectively, of the squamous lung cancer cell lines HCC-95 and HCC-1588 showing partially positive CK7 (more than in 2D cultures for HCC-1588, see Suppl Fig 2) and negative TTF-1. The scale bar is 20 µm.

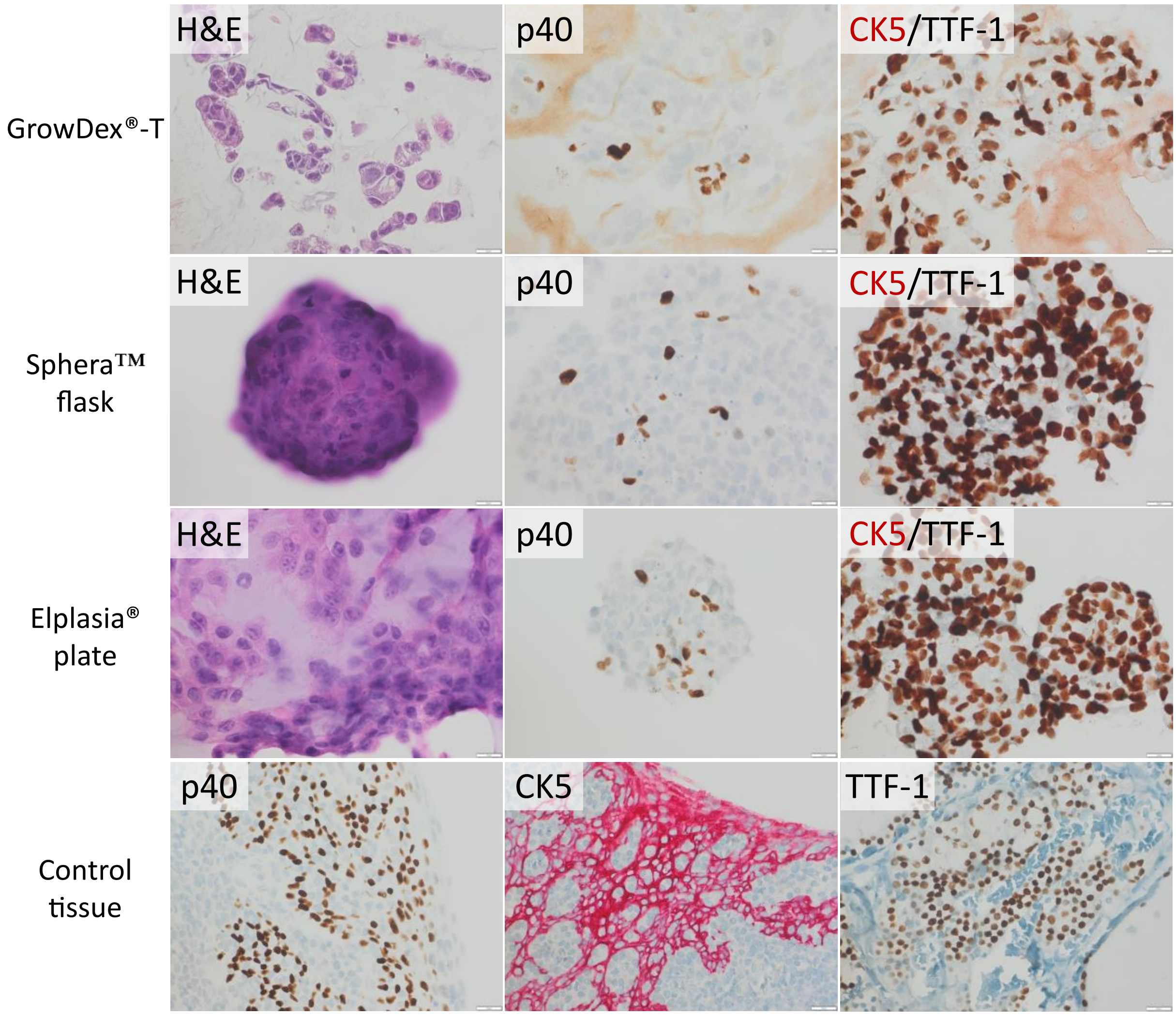

Supplementary Figure 6. Staining of 3D tumoroid cultures from GrowDex®-T hydrogel, Nunclon™ Sphera™ flasks, and Corning® Elplasia® 6 well plates, respectively, of the lung adenocarcinoma cell line NCI-H1975 showing scattered p40-positive cells (as in 2D cultures, see Suppl Fig 2) and negative CK5. Note that Red Chromogen was used for the CK5 staining as part of double staining with TTF-1 (which is positive). Staining of control tissue from the slides is included for comparison (benign tonsil for p40 and CK5, and thyroid for TTF-1). The scale bar is 20 µm.

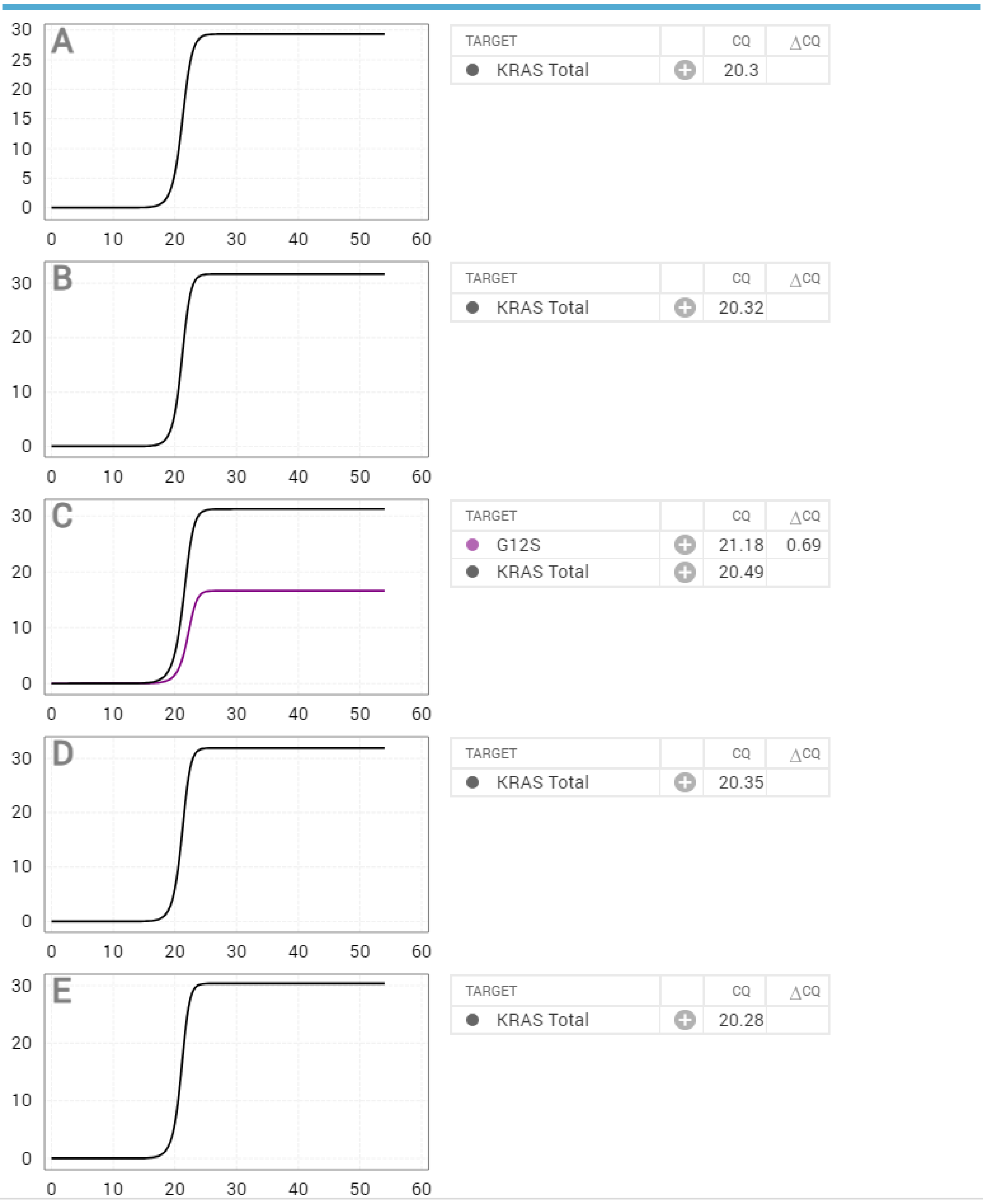

Supplementary Figure 7. Representative PCR curves from the *KRAS* analyses of the A549 cultures with detected G12S mutation (the 2D and all 3D cultures [GrowDex®-T hydrogel, Nunclon™ Sphera™ flasks, BIOFLOAT™ 96 well plates, and Corning® Elplasia® 6 well plates] showed the same result). The different chambers have primer mixes for identification of the most common *KRAS* mutations: chamber A, G12C, G12V, G13D, and Q61H/H2; chamber B, G12D, G12R, K117N1/N2, and Q61K/K2; chamber C, G12A, G12S, and Q61R/L; chamber D, A146T/V/P; chamber E, A59E/G/T.

NCI-H1975	L858R (Ct)	T790M (Ct)	Control
2D culture (reference)	25.81	26.17	
GrowDex®-T	28.63	29.88	25.33
Sphera™ flask	24.88	25.34	
BIOFLOAT™ plate	25.03	25.43	
Elplasia® plate	24.83	25.06	

Supplementary Table 1. Cycle threshold (Ct) values from the Therascreen EGFR RGQ PCR analysis for the *EGFR* mutations L858R and T790M in the DNA samples from 2D cultures of lung adenocarcinoma cell line NCI-H1975 and 3D cultures generated through the four used methods GrowDex®-T hydrogel, Nunclon™ Sphera™ flask, BIOFLOAT™ 96 well TC plates, and Elplasia® 6 well plate.
